# Cross Talk between the Calcium-Sensing Receptor and the Vitamin D System in Prevention of Cancer

**DOI:** 10.3389/fphys.2016.00451

**Published:** 2016-10-18

**Authors:** Abhishek Aggarwal, Enikö Kállay

**Affiliations:** ^1^Department of Pathophysiology and Allergy Research, Center of Pathophysiology, Infectiology and Immunology, Medical University of ViennaVienna, Austria; ^2^Department of Pediatrics/Endocrinology, School of Medicine, Stanford UniversityStanford, CA, USA

**Keywords:** CaSR, VDR, VDRE, colorectal cancer, calcium, vitamin D

## Abstract

There is epidemiological evidence for the cancer preventive effect of dietary calcium (Ca^2+^) and vitamin D. This effect is strongest in colorectal cancer (CRC). The active vitamin D metabolite, 1,25-dihydroxyvitamin D_3_ (1,25D_3_), bound to its receptor, the vitamin D receptor (VDR) regulates the expression of hundreds of different genes in a cell- and tissue-specific manner. While Ca^2+^ acts through multiple mechanisms and pathways, some of its effects are mediated by the calcium-sensing receptor (CaSR). The joint action of Ca^2+^ and 1,25D_3_ is due to the fact that both regulate some of the main processes involved in the development of various cancers, such as proliferation, differentiation, apoptosis, migration, and inflammation. Moreover, 1,25D_3_, bound to VDR can induce translation of the CaSR, while the amount and activity of the CaSR affects 1,25D_3_ signaling. However, the complexity of the cross-talk between the CaSR and the vitamin D system goes beyond regulating similar pathways and affecting each other's expression. Our aim was to review some of the mechanisms that drive the cross-talk between the vitamin D system and the CaSR with a special focus on the interaction in CRC cells. We evaluated the molecular evidence that supports the epidemiological observation that both vitamin D and calcium are needed for protection against malignant transformation of the colon and that their effect is modulated by the presence of a functional CaSR.

## Introduction

Epidemiological and preclinical studies suggested that dietary calcium and vitamin D are able to prevent several forms of cancer, with strongest effect observed in prevention of colorectal cancer (CRC; Zhang and Giovannucci, 2011). Low calcium intake and vitamin D insufficiency were considered independent risk factors for cancer, until Garland et al. showed that their colon cancer preventing effect is interdependent (Garland et al., 1985). Both calcium and vitamin D affect several hallmarks of cancer: enhance differentiation, adhesion, activate apoptosis, inhibit proliferation and inflammation, and decrease metastatic potential. Therefore, understanding the interactions between them would enhance the possibility of exploiting their cancer preventing potential.

### Epidemiological evidence

Low calcium intake was linked to the pathogenesis of several chronic diseases and is a recognized risk factor for total cancer incidence (Park et al., 2009; Peterlik et al., 2013). In a population-based, double-blind, placebo-controlled randomized trial among community-dwelling women dietary calcium (1400–1500 mg) and vitamin D (1100 IU) reduced all-cancer risk (Lappe et al., 2007). Calcium and vitamin D supplementation reduced melanoma risk in women with a history of non-melanoma skin cancer (Tang et al., 2011). High serum calcium levels at baseline were associated with lower breast cancer mortality in a Swedish cohort, while serum 25 hydroxyvitamin D_3_ (25D_3_) levels and breast cancer mortality showed a u-shaped correlation (Luo et al., 2013; Huss et al., 2014). A recent study showed that high calcium-sensing receptor (CaSR) expression in primary prostate tumors was associated with lethal progression of the disease if the tumors expressed low vitamin D receptor (VDR) levels, but not if the tumors had high VDR levels (Ahearn et al., 2016).

Expression level and activity of the CaSR were linked to risk, incidence, recurrence, or lethality of various cancers, such as prostate, breast, colorectal, ovarian cancer, or neuroblastoma (Tennakoon et al., 2016), cancers where vitamin D insufficiency might also be involved in etiology (Table 1).

**Table 1:** **Effect of calcium, vitamin D and involvement of the CaSR in development of cancer**.

**Cancer type**	**Effect of calcium**	**Effect of vitamin D**	**CaSR involvement**
Parathyroid adenoma/Carcinoma	↓ Proliferation	↓ Proliferation	Tumor suppressor (Miller et al., 2012)
Colorectal cancer	↓ Incidence	↓Incidence^*^	Tumor suppressor (Aggarwal et al., 2015b)
	↓ Risk	↓*Risk*	
Neuroblastoma			Tumor suppressor (Casala et al., 2013)
Breast cancer	↓*Cancer risk*	↓*Incidence*	↑ Metastasis to bone (Vanhouten and Wysolmerski, 2013)
		↓*Risk*	
		*↑ Survival*	
Prostate cancer	*↑ Cancer incidence*	*↑ Protection against aggressive cancer*	↑ Metastasis to bone (Liao et al., 2006; Chakravarti et al., 2009)
Kidney cancer			↑ Metastasis to bone (Joeckel et al., 2014)

* *Italics: limited evidence*.

In CRC there is ample evidence supporting the cancer preventive effects of calcium and vitamin D (World Cancer Research Fund/American Institute for Cancer Research, 2007; Tarraga Lopez et al., 2014). In adenomatous polyposis patients high doses of dietary calcium and vitamin D significantly reduced the rate of polyp formation after 6 months (Holt et al., 2006). Pooling data from 10 cohort studies showed that only high intake of both vitamin D and calcium reduced risk of CRC (Cho et al., 2004). Calcium supplementation in a placebo-controlled randomized multi-center clinical trial reduced risk of adenoma recurrence (RR = 0.71) only in subjects with 25D_3_ levels above the median (29.1 ng/mL). Moreover, serum 25D_3_ levels correlated inversely with adenoma risk only in subjects receiving calcium supplementation (Grau et al., 2003). Usually changes in serum Ca^2+^ levels are minimal; however dietary calcium causes high fluctuations in fecal Ca^2+^ levels that affect tumorigenesis. Ca^2+^ in the intestine forms insoluble salts with potentially carcinogenic bile acids, such as deoxycholic and lithocholic acid. Lithocholic acid is able to bind VDR, induce expression of the vitamin D degrading enzyme (CYP24A1) and 1,25 dihydroxyvitamin D_3_ (1,25D_3_) degradation (Hobaus et al., 2013a).

While interventional studies are sometimes inconclusive, many animal studies convincingly demonstrated the cancer-preventing effect of vitamin D_3_ and calcium, suggesting that calcium and vitamin D regulate the dynamic balance between proliferation, differentiation, adhesion, motility, and apoptosis in the colon (Holt et al., 2006; Hummel et al., 2013) and both are needed for optimal effectiveness. In a dietary colon cancer model, where the so-called new western diet (NWD) fed for 2 years led to development of spontaneous colorectal tumors, supplementation with dietary calcium and vitamin D reduced significantly both colon tumor incidence and multiplicity (Newmark et al., 2009). Moreover, the high Ca^2+^ and vitamin D intake prevented colonic tumor formation even in the mice bearing the Apc1638N^+/−^ mutation and fed the NWD (Yang et al., 2008). How far the effect of calcium was mediated by the CaSR is not yet clear.

### Mechanism of action

The mechanism of action of vitamin D is clear: vitamin D (synthesized in the skin or ingested through food) is transformed in the liver to 25 hydroxyvitamin D_3_ and in the kidney (or other tissues), to its most active form 1,25 dihydroxyvitamin D_3_. The enzymes involved in this process are the vitamin D_3_ 25 hydroxylases (e.g., CYP2R1 and CYP27A1) and the 25 hydroxyvitamin D_3_ 1 alpha hydroxylase (CYP27B1). 1,25D_3_ binds to its nuclear receptor, the transcription factor VDR and regulates target gene expression. Both 1,25D_3_ and its precursor 25D_3_ are catabolized by the 1,25 dihydroxyvitamin D_3_ 24 hydroxylase (CYP24A1) (Christakos et al., 2016). VDR, CYP27B1, and CYP24A1 are almost ubiquitously expressed, suggesting that 1,25D_3_ can be synthesized and degraded in most of the tissues. Effectiveness of 1,25D_3_ depends on the local expression level and activity of all these molecules.

The mechanism of action of extracellular calcium is by far much more complex. There are numerous different molecules (different types of Ca^2+^ channels, Ca^2+^ pumps, exchangers, etc.) and pathways that are involved (Capiod, 2016). The extracellular calcium-sensing receptor (CaSR) is one of the candidates that mediate the cancer preventive effects of dietary calcium (Tennakoon et al., 2016). Whitfield suggested that the CaSR serves as the molecular switch that turns on differentiation and turns off proliferation in colonic epithelial cells as these migrate along the colonic crypt (Whitfield, 2009).

The main physiological role of this G protein-coupled receptor is regulating calcium homeostasis (Brown et al., 1993) by maintaining the balance between Ca^2+^ absorption in the intestine, Ca^2+^ excretion by the kidneys, and the release of Ca^2+^ from bone. The maintenance of calcium homeostasis is orchestrated by the intricate cross talk among Ca^2+^, the CaSR, parathyroid hormone (PTH), and the active vitamin D hormone 1,25D_3_ (Brown and MacLeod, 2001). The CaSR regulates PTH secretion, dependent on extracellular Ca^2+^ concentrations. When Ca^2+^ levels are high in the serum, the receptor is activated, leading to a decrease in PTH synthesis and secretion. When serum Ca^2+^concentration drops, CaSR is inactive, and PTH is secreted into the serum, leading to increased urinary Ca^2+^ resorption, stimulation of 1,25D_3_ production in the kidney, which enhances Ca^2+^ uptake from the intestine and calcium release from the bone. As an effect of this, serum Ca^2+^ concentration is increased again, activating the receptor and the cycle continues. This function is described in more detail elsewhere in this Research Topic (Roszko et al., 2016; Conigrave, in review).

The CaSR is expressed not only in calciotropic organs, such as the parathyroid, kidney, bone, and small intestine, but also in several organs not directly involved in maintaining calcium homeostasis. In these tissues, the CaSR is involved in a multitude of cellular processes including secretion, chemotaxis, cell–cell adhesion, and control of proliferation, differentiation, and apoptosis (Brown and MacLeod, 2001; Brennan et al., 2013).

The CaSR and the vitamin D system cooperate not only in calciotropic organs, but also in the skin (Tu and Bikle, 2013), in the colon (Canaff and Hendy, 2002; Chakrabarty et al., 2005; Aggarwal et al., 2016), and probably in other tissues as well. The cross-talk between CaSR and vitamin D in the skin is presented in detail by Bikle and colleagues in this Research Topic (Bikle et al., 2016). Therefore, this review concentrates mainly on the interdependence of CaSR and vitamin D signaling in the colon and focuses on the CaSR-mediated crosstalk between Ca^2+^ and vitamin D.

## Effect of vitamin D on CaSR expression

The CaSR and the vitamin D system become deregulated during tumorigenesis through different mechanisms. CaSR expression is lost in colorectal tumors (Sheinin et al., 2000; Chakrabarty et al., 2005; Fetahu et al., 2014a), mainly due to epigenetic silencing i.e., DNA hypermethylation, histone deacetylation, increased expression of the mircroRNAs miR-135b, miR-146b (Hizaki et al., 2011; Fetahu et al., 2014a, 2016). In unfavorable neuroblastomas CaSR is silenced by epigenetic and genetic mechanisms (Casala et al., 2013), while in parathyroid tumors CaSR loss is independent of DNA methylation (Varshney et al., 2013).

Shortly after the discovery of the CaSR, it was shown that on one hand, in vitamin D-depleted rats the CaSR expression was significantly reduced; on the other hand, intraperitoneal administration of 1,25D_3_ upregulated parathyroid, thyroid, and kidney CaSR expression (Canaff and Hendy, 2002). We have shown that dietary vitamin D was able to upregulate CaSR expression also in the colon of mice (Aggarwal et al., 2016). *In vitro* 1,25D_3_ increased CaSR expression in a thyroid C cell line, in the proximal tubule human kidney cells (HKC) (Canaff and Hendy, 2002), and in colon cancer cells (Chakrabarty et al., 2005; Fetahu et al., 2014b). An essential prerequisite for the direct modulation of transcription by 1,25D_3_ is the location of at least one liganded VDR protein close to the transcriptional start site (TSS) of the primary target gene. It was Canaff and her colleagues who have demonstrated that the *CaSR* gene has two functional promoters (P1 and P2), and both contain a vitamin D response element (VDRE) upstream of the TSSs (Canaff and Hendy, 2002).

Both VDREs are often methylated in colon cancer (Fetahu et al., 2014b), and the level of silencing of the CaSR varies depending on the level of DNA methylation and of histone acetylation at distinct residues. The epigenetic landscape of the CaSR promoter affects also its transcriptional and translational upregulation by 1,25D_3_ (Fetahu et al., 2014b). In two colon cancer cell lines expressing undetectable levels of CaSR 1.4 mM Ca^2+^ or 1 μM 1,25D_3_ were able to reduce CaSR promoter methylation and thus contribute to the upregulation of CaSR expression (Singh et al., 2015). Whether high dietary vitamin D and calcium would reduce or prevent methylation of the CaSR promoter also *in vivo* needs to be tested, as 1 μM concentrations of 1,25D_3_ in the tumor microenvironment would be difficult to obtain.

## Effect of the CaSR on expression of the vitamin D system

Although the kidney is the main source of serum 1,25D_3_ levels, the extra-renally synthesized 1,25D_3_, which acts locally in an autocrine and paracrine manner, is an indispensable source for the cancer-preventive action of vitamin D. However, during tumor development the expression of the different molecules of the vitamin D system in the affected tissue becomes deregulated. In undifferentiated colorectal adenocarcinomas not only CaSR expression, but also expression of VDR and CYP27B1 is lower than in differentiated tumors (Bareis et al., 2002; Bises et al., 2004; Giardina et al., 2015). Whether these phenomena are linked or not, needs to be determined. Nevertheless, loss of CaSR expression in an epidermis-specific CaSR knock-out mouse model led to significantly lower vdr and cyp27b1 expression in the skin compared with the wild type controls (Tu et al., 2012), suggesting that intact CaSR expression and function is needed for proper expression of the vitamin D system.

One of the causes of VDR loss in colorectal tumors is the increased expression of the transcription factor SNAIL1, one of the main regulators of the epithelial-to-mesenchymal transition (Palmer et al., 2004). Finding ways to prevent SNAIL1 upregulation would prevent VDR loss and preserve sensitivity to the anti-proliferative effects of 1,25D_3_. We were able to show that transfection of the HT29 colon cancer cell line with the functional CaSR prevented epithelial-to-mesenchymal transition and upregulation of SNAIL1. Similar effects were seen by activating the receptor with the allosteric CaSR activator NPS-R568 (Aggarwal et al., 2015a).

In colorectal tumors the expression of the vitamin D degrading enzyme, CYP24A1 is significantly higher when compared with the adjacent normal tissue (Horvath et al., 2010). This higher expression was due, at least in part, to more copies of the *CYP24A1* gene. In these tumors, CYP24A1 expression correlated with proliferation rate of the tumors (Hobaus et al., 2013b). CYP24A1 overexpression conferred a more aggressive growth to colon cancer tumor xenografts (Hobaus et al., 2016). In human patients CYP24A1 has been suggested to be a biomarker for progression and prognosis of CRC (Sun et al., 2016). Upregulation of CYP24A1 is common in different solid tumors, such as lung, prostate, breast, cervical, ovary tumors (Anderson et al., 2006; Luo et al., 2013; Ahn et al., 2016). In these tumors, CYP24A1 reduces the half-life of 1,25D_3_, shortens the duration of the vitamin D signal, and thus reduces the anti-tumorigenic effects of the active 1,25D_3_. We have shown previously that low dietary calcium intake (0.04%) increased CYP24A1 expression in the colon of mice, while high calcium (0.9%) prevented CYP24A1 increase (Kállay et al., 2005). Whether the CaSR mediated this effect was not demonstrated, but our observation that in the colon of CaSR and PTH double knock-out mice cyp24a1 expression was higher compared with wild-type mice suggests a contribution of the CaSR.

## Effect of CaSR expression and functionality on vitamin D action

The complexity of the cross-talk between the CaSR and the vitamin D system goes beyond affecting expression mutually. In CRC cells the anti-tumorigenic effects of 1,25D_3_ are influenced by the amount of CaSR expressed in a cell and by the activity of this receptor (Singh et al., 2013; Aggarwal et al., 2016). Liu et al. (2010) have shown that 1 μM 1,25D_3_ reduced cell number and inhibited invasion only in cells expressing the CaSR, but not in cells where the CaSR expression was down-regulated by transfection with CaSR-specific shRNA. In Caco-2 cells, transfection of a mutated CaSR harboring the inactivating mutation R185Q, abolished the anti-proliferative effect of 1,25D_3_ while the overexpression of the wild-type CaSR significantly intensified the anti-proliferative 1,25D_3_ effect. Treating the cells with the allosteric CaSR activator NPS R-568 increased the effect of 1 nM 1,25D_3_ on cell number even in the cells expressing the mutant CaSR (Aggarwal et al., 2016).

**Figure 1:**
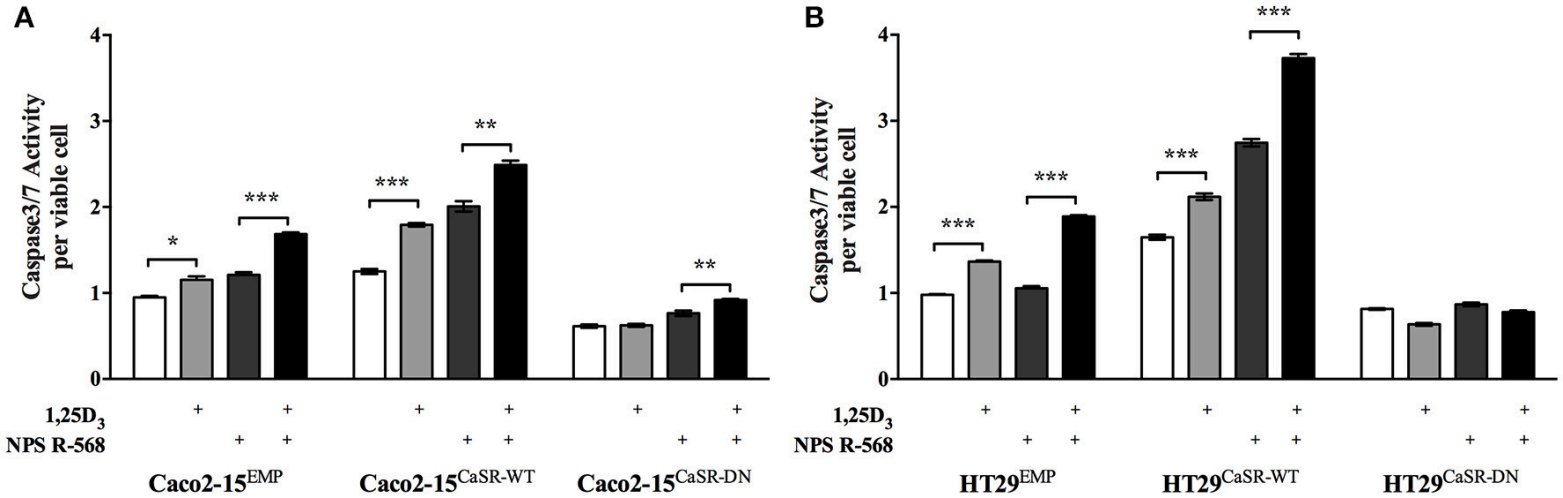
**Impact of the inactivating CaSR mutation on the apoptotic effect of 1,25D_**3**_ and NPS R-568 in the colon cancer cell lines Caco2-15 (A) and HT29 (B), stably transfected with wild type (CaSR-WT) or the dominant negative R185Q mutant (CaSR-DN)**. Cells transfected with and empty vector (EMP) were used as controls. One week post confluence, the cells were treated with 1,25D_3_ (1 nM), NPS R-568 (1 μM) or a combination of 1,25D_3_ and NPS R-568 for 48 h. Apoptosis was assessed by measuring caspase3/7 activity. Bars represent mean ± *SEM*. Statistical significance was calculated using two-way ANOVA followed by Tukey's correction. ^*^p < 0.05, ^**^p < 0.01, ^***^p < 0.001. Modified from Aggarwal et al. (2016).

1,25D_3_ has strong apoptosis-promoting effects, similar to Ca^2+^. We have shown that 1 nM 1,25D_3_ effectively induced caspase 3/7 activity in colon cancer cells in the presence of 1.8 mM Ca^2+^. This effect was significantly stronger when the cells overexpressed the wild type CaSR. NPS R-568 treatment almost doubled the effect of Ca^2+^ in the cells overexpressing the CaSR and this effect was further enhanced by 1,25D_3_. The inactivating CaSR mutant (R185Q) significantly reduced the apoptotic effect of 1,25D_3_ (Figure 1) (Aggarwal et al., 2016), although the mechanism is not clear. Inactivating mutations of the CaSR can cause disorders of calcium metabolism including familial hypocalciuric hypercalcemia type 1 (FHH1) and neonatal severe hyperparathyroidism (NSHPT). The impact of CaSR mutations on the colon physiology is not known. To date, no CaSR mutations have been associated with CRC risk or mortality, although there are suggestions that certain polymorphisms (including A986S, R990G, Q1011E) could be linked to CRC risk (Dong et al., 2008; Jacobs et al., 2010).

One apoptosis-inducing mechanism of 1,25D_3_ is the upregulation of the expression of the inducers of cell cycle arrest and apoptosis: the cyclin-dependent kinase inhibitors Cdkn1a and Cdkn1b (Chu et al., 2008; Karimian et al., 2016). In a colon cancer cell line the effect of 1,25D_3_ on Cdkn1a was abolished by knocking down the CaSR by CaSR-shRNA transfection. Moreover, the expression of survivin, a key anti-apoptotic protein, and the activity of the survivin promoter was inhibited by 1,25D_3_ only in cells expressing the wild-type CaSR and not in cells transfected with CaSR-shRNA (Liu et al., 2010).

Overexpression of the CaSR prevented the mesenchymal transition and the acquisition of stem cell-like properties in the HT29 colon cancer cell line (Aggarwal et al., 2015a) reducing the metastatic properties of the cells. Another mechanism by which CaSR might regulate adhesion and migration is the regulation of the activity of integrins, adhesion receptors that mediate cell migration (Tharmalingam and Hampson, 2016). Interestingly, 1,25D_3_ also affects integrin signaling, as it has been shown that 1,25D_3_ inhibited ionizing radiation-mediated upregulation of several integrins in keratinocytes (Muller et al., 2006).

Colon cancer cells transfected with CaSR express lower levels of thymidylate synthase (TS) (Liu et al., 2010), an enzyme involved in *de novo* DNA synthesis which is the target of the main colon cancer drug 5-fluoro uracil (5-FU) (Chu et al., 2003). 1,25D_3_ further inhibits TS expression and promoter activity and thus strengthens the cytotoxic 5-FU effect, however only in the cells expressing endogenous CaSR but not if the CaSR was knocked down (Liu et al., 2010). These data warrant for further studies to explore the possibility of using 1,25D_3_ and calcimimetics in supporting combinatorial anticancer therapy.

## Interplay between CaSR and 1,25D_3_ signaling in regulating the Wnt pathway

In a recent clinical trial, supplementation of the Western-style diet with 1,25D_3_ (0.5 μg/day) with or without 2 g calcium showed that in human colorectal mucosa 1,25D_3_ upregulated expression of multiple genes, such as those involved in inflammation and immunity, while Ca^2+^ modulated this effect (Protiva et al., 2016). The joint action of Ca^2+^ and 1,25D_3_ is due to the fact that both regulate some of the main processes involved in the development of various cancers. Among the best characterized pathways involved in many of these processes is the Wnt pathway.

Wnt proteins are secreted glycoproteins that signal by interacting with the receptors called Frizzled and Lipoprotein-related peptide 5/6 to release ß-catenin from its destruction complex. Colon cancer is regarded as a disease of defective Wnt-signaling (Gregorieff and Clevers, 2005; Clevers, 2006; Cancer Genome Atlas Network, 2012). The adenomatous polyposis coli (APC), a member of the ß-catenin destruction complex, is one of most often mutated components of the canonical Wnt-signaling (Powell et al., 1992) and leads to accumulation of ß-catenin and constitutive activation of the Wnt pathway (Behrens, 2005). The activated Wnt pathway induces proliferation by upregulating expression of target genes involved in DNA replication, cell cycling, such as cell division cycle 6 (CDC6), mini chromosome maintenance (MCM) 3/5, cyclin D1, c-myc. Both calcium and vitamin D regulate the Wnt pathway (Palmer et al., 2001; Shah et al., 2006; Cross and Kallay, 2009), usually inhibiting the canonical and activating the non-canonical Wnt pathway (Macleod, 2013).

In the intestine the CaSR expression inversely correlated with activity of the canonical Wnt/ß-catenin pathway. Loss of CaSR increased ß-catenin phosphorylation on Ser-552 and Ser-675 residues, which led to increased nuclear localization of ß-catenin in colonocytes and promoted its transcriptional activity (Rey et al., 2012). In colorectal tumors CaSR expression inversely correlated with expression of several proliferation markers, such as CDC6, MCM2, MCM5, MCM6, and these markers were also reduced in the colon of mice lacking the CaSR (Aggarwal et al., 2015b). Increasing CaSR expression in colon cancer cell lines led to reduced nuclear translocation of ß-catenin and to higher E-cadherin protein levels (Aggarwal et al., 2015a). In the CBS CRC cell line 1 mM Ca^2+^ alone, or in combination with 100 nM 1,25D_3_ inhibited the Wnt/ß-catenin pathway by upregulating E-cadherin and inhibiting T-cell factor 4 (TCF-4) expression (Chakrabarty et al., 2005). E-cadherin is a type-1 transmembrane protein that binds ß-catenin thus sequestrating it to the membrane and preventing its nuclear translocation, while TCF-4 is the transcription factor that together with ß-catenin regulates the transcription of the target genes of the Wnt pathway (http://web.stanford.edu/group/nusselab/cgi-bin/wnt/target_genes).

In myofibroblasts, activation of CaSR with various agonists, such as Ca^2+^, poly-L-Arginine, spermine, or neomycin induced secretion of the Wnt antagonists Dickkopf 1 (Dkk-1) and Wnt5a. This could be prevented by transient transfection of the cells with CaSR-specific siRNA. In colon cancer cells CaSR activation increased expression of the low density lipoprotein receptor-related protein 6 (Lpr6). Dkk-1 bound to Lpr6 upregulated E3 ubiquitin ligase expression leading to degradation of ß-catenin and thus to inhibition of Wnt signaling (MacLeod et al., 2007; Macleod, 2013). Moreover, activation of the CaSR upregulated the Wnt5a receptor, the receptor tyrosine kinase-like orphan receptor 2 (Ror-2), leading to reduced expression of the tumor necrosis alpha receptor (Kelly et al., 2011).

1,25D_3_ regulates Wnt signaling also by multiple mechanisms, some very similar to CaSR effects. In colon cancer cells 1,25D_3_ inhibited TCF-4 transcription, upregulated expression of E-cadherin and of other adhesion proteins, and induced nuclear export of ß-catenin. The direct binding of the liganded VDR to ß-catenin inhibited its interaction with TCF-4 (Palmer et al., 2001). 1,25D_3_ was also able to upregulate DKK-1 expression in colon cancer cell lines similar to the effect of CaSR in myofibroblasts (Pendas-Franco et al., 2008).

Thus, functional CaSR and vitamin D system can reduce the impact of an overactive Wnt pathway overcoming, at least in part, the loss of APC activity.

## Conclusions

**Figure 2:**
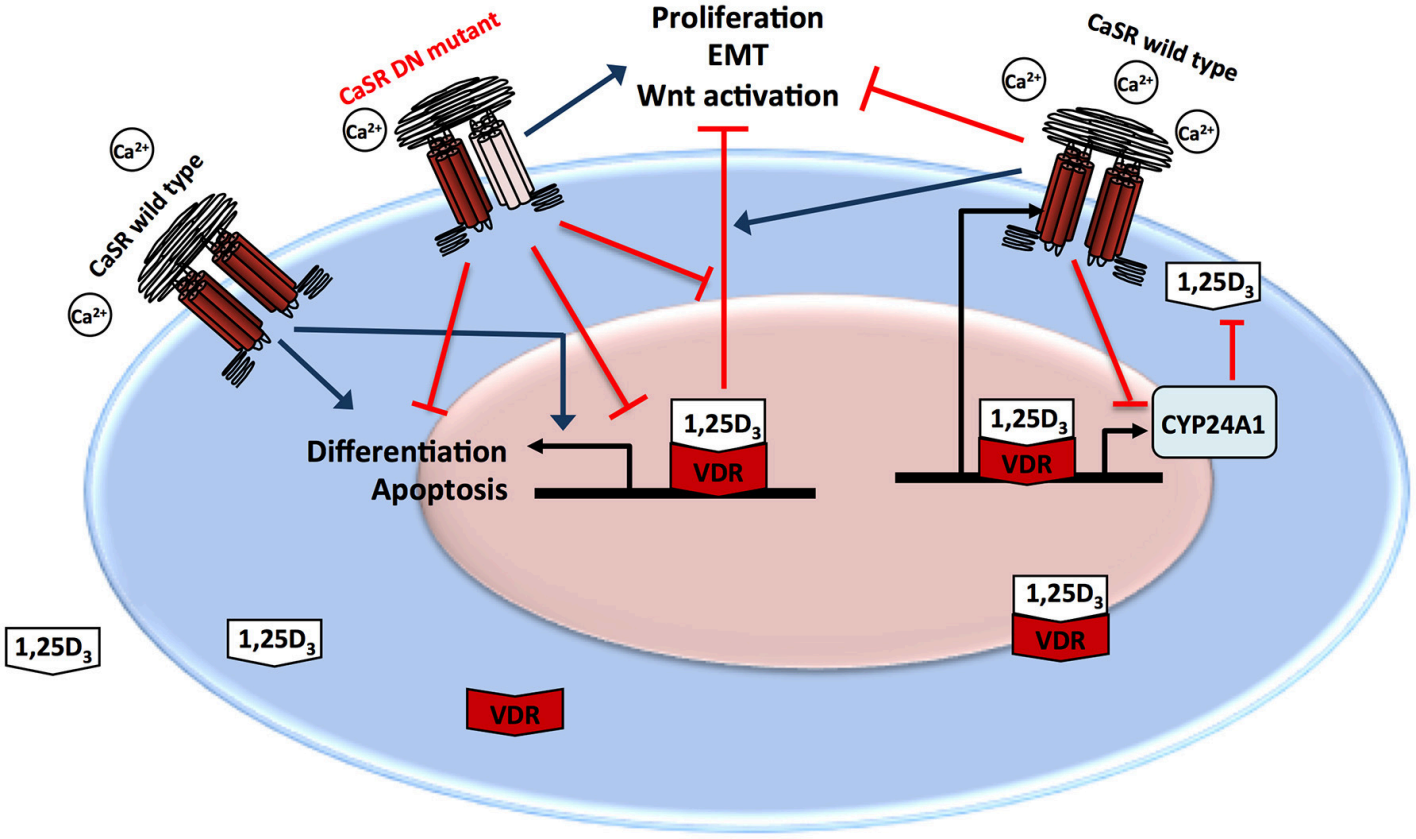
**Interactions between the vitamin D system and the CaSR**. Ca/CaSR and the 1,25D_3_/VDR cross talk to protect colonic epithelial cells from malignant transformation. 1,25D_3_ is able to up regulate expression of both, CaSR and Cyp24a1. The wild type CaSR has a tumor suppressive role in the colon promoting (blue arrows) differentiation and apoptosis and suppressing (red arrows) proliferation and EMT and potentiates the tumor preventive effects of 1,25D_3_. The presence of a DN mutant CaSR abrogates the tumor preventive effects of both Ca and 1,25D_3_.

This review provides molecular evidence for the interaction between the vitamin D system and the CaSR (Figure 2). It supports the epidemiological observation that vitamin D and calcium are both needed to protect against malignant transformation, at least in the colon and that their effect depends, at least in part from the presence of a functional CaSR.

It is feasible that in NSHPT or FHH1 patients with inactivating CaSR mutations vitamin D is less effective in regulating proliferation, differentiation, and apoptosis. These patients might therefore have a higher risk to develop cancer. The anti-proliferative effects of 1,25D_3_ should be studied also in the parathyroid of FHH1 patients. The marked parathyroid hyperplasia in FHH1 or NSHPT might be caused both by the less effective CaSR and by loss of the anti-proliferative function of 1,25D_3_ due to mutated CaSR.

## Author contributions

EK and AA have written the manuscript. AA has made the figures.

### Conflict of interest statement

The authors declare that the research was conducted in the absence of any commercial or financial relationships that could be construed as a potential conflict of interest. The reviewer KMD and handling Editor declared their shared affiliation, and the handling Editor states that the process nevertheless met the standards of a fair and objective review.
